# Examining buprenorphine compared to methadone for likelihood of overdose during pregnancy, 2014–2021

**DOI:** 10.1002/pmf2.70276

**Published:** 2026-04-07

**Authors:** Amy Board, Alana M. Vivolo‐Kantor, Shin Y. Kim, Shawn A. Thomas, Emmy L. Tran, Mishka Terplan, Pilar M. Sanjuan, Marcela C. Smid, Tanner Wright, Neil S. Seligman, Patrick D. Schneider, Autumn Davidson, Michelle L. Henninger, Elisha M. Wachman, Kara M. Rood, Luigi Garcia Saavedra, Judette Louis, Diane Morse, Julie Shakib, Kelley A. Saia, Kathryn Miele

**Affiliations:** ^1^ Division of Birth Defects and Infant Disorders National Center on Birth Defects and Developmental Disabilities, Centers for Disease Control and Prevention Atlanta Georgia USA; ^2^ Division of Overdose Prevention National Center for Injury Prevention and Control, Centers for Disease Control and Prevention Atlanta Georgia USA; ^3^ Epidemic Intelligence Service, Centers for Disease Control and Prevention Atlanta Georgia USA; ^4^ Friends Research Institute Baltimore Maryland USA; ^5^ Department of Family and Community Medicine University of New Mexico Health Sciences Center Albuquerque New Mexico USA; ^6^ University of Utah Health Salt Lake City Utah USA; ^7^ University of South Florida Tampa Florida USA; ^8^ University of Rochester School of Medicine Rochester New York USA; ^9^ The Ohio State University Columbus Ohio USA; ^10^ Kaiser Permanente Portland Oregon USA; ^11^ Center for Health Research—Kaiser Permanente Northwest Portland Oregon USA; ^12^ Department of Pediatrics Boston Medical Center Boston Massachusetts USA; ^13^ New Mexico Department of Health Substance Use Epidemiology Section Albuquerque New Mexico USA

**Keywords:** buprenorphine, medication for opioid use disorder, methadone, opioid use disorder, overdose, pregnancy

## Abstract

**Objective:**

To examine the likelihood of experiencing an overdose among women receiving any buprenorphine (with or without naloxone) compared to women receiving methadone during pregnancy.

**Methods:**

Data from the MATernaL and Infant clinical NetworK (MAT‐LINK), a longitudinal surveillance system of pregnant woman–child dyads using electronic health records (EHRs), were analyzed. Women from geographically diverse US clinical sites who had a diagnosis of opioid use disorder (OUD) during pregnancy, were prescribed medication for opioid use disorder (MOUD), and had a known birth outcome occurring between January 1, 2014 and August 31, 2021 were included. The Andersen–Gill Cox regression model for recurrent events with robust standard errors was used to model the effect of MOUD type by trimester on time to overdose or date of pregnancy outcome and calculate adjusted hazard ratios (aHRs).

**Results:**

Among the 3682 pregnancies included in this analysis, 46 (1.2%) had at least one EHR‐documented overdose. None of the overdoses were fatal. In adjusted models, no differences in overdose were observed between pregnancies in which buprenorphine was prescribed or administered compared to pregnancies in which methadone was prescribed or administered (any drug overdose: aHR, 0.62; 95% CI, 0.31–1.23; opioid‐involved overdose: aHR, 0.49; 95% CI, 0.18–1.30). In a trimester‐specific sub‐analysis of pregnancies with at least two MOUD prescriptions or administrations, taking buprenorphine in the third trimester resulted in a decreased likelihood of any drug overdose (aHR, 0.43; 95% CI, 0.20–0.93) and opioid‐involved overdose (aHR, 0.32; 95% CI, 0.11–0.92) compared with methadone.

**Conclusion:**

It is important that women with OUD are initiated on the medication that works best for them as early as possible during pregnancy and that reinforcement of the importance of MOUD occurs throughout pregnancy, paying special attention to disruptions in treatment during the third trimester that might increase risk for overdose.

## 1 INTRODUCTION

Drug overdose rates among pregnant women increased at an alarming pace over the past decade. From 2017 to 2020, the pregnancy‐associated overdose mortality rate increased by 81% from 6.56 to 11.85 per 100,000 [1], and pregnancy‐associated overdose mortality more than tripled among perinatal women aged 35 to 44 years from 2018 to 2021 (from 4.9 to 15.8 per 100,000 mothers) [2]. Nonfatal overdose is also common among women with opioid use disorder (OUD) during pregnancy; one study of pregnant women with severe OUD found that nearly two‐thirds reported ever experiencing an overdose, with 41.2% overdosing in the past year [3].

Medications for opioid use disorder (MOUDs), including buprenorphine (with or without naloxone) and methadone, are recommended for women with OUD during pregnancy by the American College of Obstetricians and Gynecologists [4, 5] and have been shown to reduce the likelihood of experiencing an overdose postpartum [6, 7]. One study also noted that longer duration of MOUD during pregnancy was associated with a significantly reduced risk of nonfatal overdose during pregnancy [8]. However, much of the literature on MOUD during pregnancy is focused on perinatal or infant outcomes such as preterm birth [9, 10, 11], neonatal abstinence syndrome (NAS) or neonatal opioid withdrawal syndrome (NOWS) [9, 10, 11, 12], and low birthweight [9, 10]. Some studies have noted differences between buprenorphine and methadone, with buprenorphine potentially yielding reduced likelihood of adverse outcomes such as preterm birth and NAS when compared with methadone [11, 13, 14]. However, few data are available on overdose during pregnancy, including differences between women receiving buprenorphine compared to those receiving methadone.

Using data from a longitudinal cohort of women with documentation of MOUD during pregnancy, this analysis examined the likelihood of experiencing an overdose during pregnancy among women prescribed buprenorphine (with or without naloxone) compared to women prescribed methadone.

## 2 MATERIALS AND METHODS

### 2.1 Study sample

The MATernaL and Infant clinical NetworK (MAT‐LINK) conducts longitudinal surveillance of pregnant woman–child dyads using electronic health records (EHRs) from geographically diverse US clinical sites. This analysis included women with a diagnosis of OUD during pregnancy (Table S1), at least one EHR‐documented MOUD during pregnancy and trimester of MOUD receipt recorded in the EHR, and a known pregnancy outcome occurring between January 1, 2014 and August 31, 2021. Pregnancies were excluded if they had a missing or incorrect estimated date of delivery or gestational age or a recorded overdose where the date the overdose occurred was missing or occurred prior to the start of pregnancy (see flow diagram, Figure S1). All pregnancies from one clinical site were excluded given a greater than 60% missingness for key analysis variables (homelessness or housing instability and incarceration). Women with multiple pregnancies during the cohort period were included more than once in the dataset (10.3% of women, with five being the maximum number of pregnancies belonging to the same woman). Additional details about MAT‐LINK have been described elsewhere [15].

### 2.2 Measures

#### 2.2.1 MOUD classification

The exposure of interest was EHR‐documented prescription or administration of MOUD during pregnancy, which included buprenorphine (with or without naloxone), methadone, and naltrexone. Any type of MOUD prescribed or administered was used to denote MOUD during pregnancy, but naltrexone was not included in modeling analysis due to small counts (*n* = 22). Buprenorphine formulations used for chronic pain management were not included. The methods for MOUD data capture, cleaning, and categorization in MAT‐LINK have been described elsewhere [16]. For this analysis, EHR documentation of prescribed or administered buprenorphine monoproduct and buprenorphine–naloxone formulations was combined into a single “any buprenorphine” category to create a more complete and robust exposure group, which was compared to methadone.

#### 2.2.2 Overdose outcome

To identify women with an overdose during pregnancy, clinical sites abstracted overdose‐related data from available outpatient and inpatient records during each pregnancy and at delivery. Sites then submitted information via Research Electronic Data Capture (REDCap) on whether an overdose occurred, the date of the overdose, the outcome of the overdose (fatal or nonfatal), and the substance(s) involved in the overdose (substances were not mutually exclusive). The REDCap form included 24 opioids and 11 non‐opioid substances (Table S2); if the substance was not included in the list, it was reported via a free‐text field. These data were reviewed by clinical subject matter experts and coded or re‐categorized into overarching substance type categories for analysis [17].

#### 2.2.3 Covariates

**FIGURE 1:**
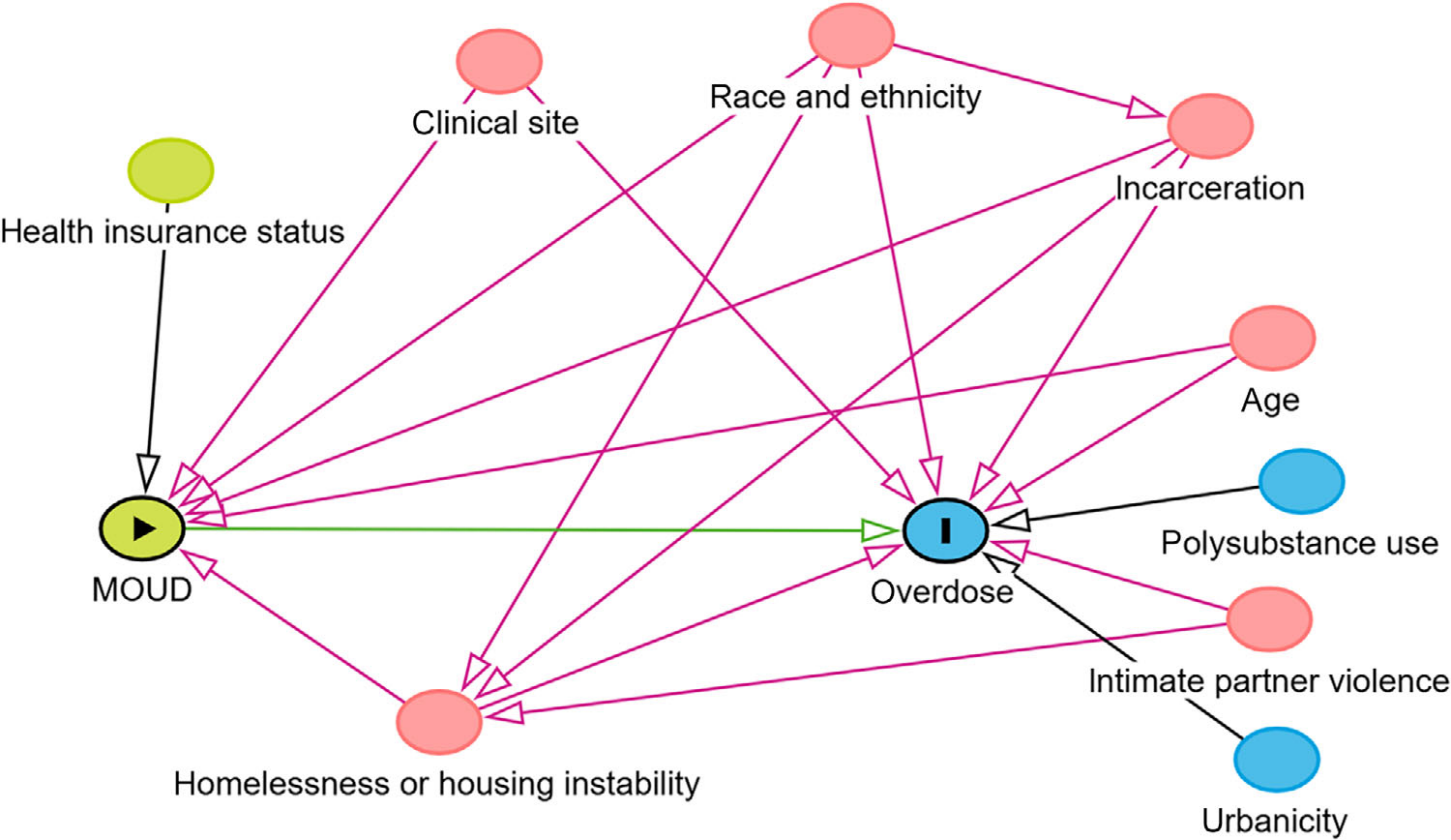
Directed acyclic graph for causal inference analysis of the impact of medication for opioid use disorder (MOUD) type on overdose during pregnancy—MATernaL and Infant clinical NetworK (MAT‐LINK), 2014–2021. The light green play button icon indicates the exposure, while the blue button with the pipe icon indicates the outcome. Light green indicates ancestors of the exposure, blue indicates ancestors of the outcome, and red indicates ancestors of the exposure and outcome. The green line is the causal path and the red lines indicate biasing paths. In this diagram, the minimal sufficient adjustment sets for estimating the total effect of MOUD type on overdose are age, race and ethnicity, clinical site, homelessness or housing instability, and incarceration during pregnancy. Diagram created using Daggity [19].

Descriptive analysis included demographic factors (age, race and ethnicity, urbanicity, health insurance status at delivery), other diagnosed substance use disorders, and psychosocial factors (intimate partner violence [IPV], homelessness or housing instability, and incarceration during pregnancy). Urbanicity was classified using Rural‐Urban Commuting Area codes, which classify ZIP codes using measures of population density, urbanization, and daily commuting, based on ZIP code at the time of delivery [18]. All other variables were from the medical record. The model included the covariates of age, race and ethnicity, homelessness or housing instability, and incarceration during pregnancy as the minimal sufficient adjustment set based on a directed acyclic graph created using Dagitty [19] (Figure 1). Clinical site was included as a fixed effect in the model to account for site‐specific variations in MOUD protocols and the availability of each type of MOUD by site.

### 2.3 Data analysis

Data cleaning was performed using Python v3.8.8 and descriptive and modeling analyses were performed using SAS v9.4 (SAS Institute). In accordance with recommended analysis approaches for repeat pregnancy outcomes [20], survey procedures were used for descriptive statistics (proc surveyfreq, using the cluster and strata options) to account for clustering of pregnancies by woman and by clinical site. Chi‐square tests were used to assess differences in characteristics among women with an overdose compared with women without an overdose during pregnancy. Because nonfatal overdose could occur more than once during pregnancy, the Andersen–Gill Cox regression model for recurrent events [21] with robust standard errors and clustering to account for multiple pregnancies belonging to the same woman was used (proc phreg with clustering options applied). Covariate‐adjusted hazard ratios (aHRs) with 95% confidence intervals (CIs) examined the effect of MOUD type by trimester on time to overdose or date of pregnancy outcome. Regression models were run to examine time to overdose for (1) the entire pregnancy, (2) the first trimester only, (3) the second trimester only, and (4) the third trimester only. Regression models were also run for any drug overdose and opioid‐involved overdoses, and were run for pregnancies with at least one MOUD prescription or administration and those with two or more MOUD prescriptions or administrations as an indicator of greater engagement in MOUD‐related care. If a woman received both buprenorphine and methadone during a trimester, this was captured in descriptive tables, but these records were dropped from the modeling analysis since the record contained data in both the exposure and the comparison group (1.2% to 3.0% of observations, depending on the regression model). Additionally, records in which the woman did not receive buprenorphine or methadone during a trimester were dropped from the modeling analysis for that trimester due to the inability to assign the record to either the exposure or comparison group (anywhere from 60.4% to 64.0% of observations in the first trimester through 7.8% to 9.9% in the third trimester).

### 2.4 Human subjects review

Because MAT‐LINK data are protected under an Assurance of Confidentiality [22], cell counts less than five were suppressed in the tables. This activity was determined to be non‐research and exempt from review by the Centers for Disease Control and Prevention's Institutional Review Board and was conducted consistent with applicable federal law and center policy (e.g., 45 CFR part 46.102.l.2; 21 CFR part 56; 42 USC §241.d; 5 USC §552a; 44 USC §3501 et seq). All MAT‐LINK clinical site activities underwent their Institutional Review Board's review and received approval or exemption prior to data collection.

## 3 RESULTS

**FIGURE 2:**
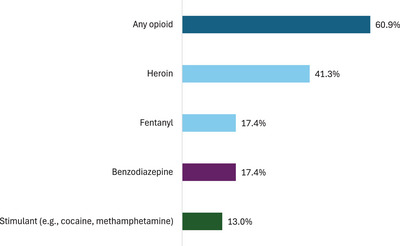
Substances Involved in overdose among women receiving medication for opioid use disorder (MOUD) during pregnancy—MATernaL and Infant clinical NetworK (MAT‐LINK), 2014–2021. *Note*: More than one substance might have been reported in an overdose event, and more than one overdose might have been reported during pregnancy. Proportions shown are calculated with the pregnancy as the unit of analysis, with 46 pregnancies in the dataset experiencing an overdose.

Among the 3682 pregnancies included in this analysis, 46 (1.2%) had at least one EHR‐documented overdose (Table 1). All overdoses were nonfatal. Among pregnancies with an overdose, the number of overdoses ranged from 1 to 4, with a median of 1. Most had an overdose involving opioids (*N* = 28, 60.9%, with 8 or 17.4% of overdoses involving fentanyl); 8 (17.4%) involved benzodiazepines and 6 (13.0%) involved stimulants (including cocaine and methamphetamine) (Figure 2). Among pregnancies with an overdose, 77.8% of women were non‐Hispanic White, compared with 62.2% of pregnancies without an overdose (*p* = 0.01) (Table 1). Additionally, more pregnancies with an overdose were among women with a diagnosed concomitant tobacco/nicotine use disorder (77.8%), stimulant use disorder (53.3%), or alcohol use disorder (13.3%), compared to pregnancies without a documented overdose (tobacco/nicotine use disorder: 62.5%, *p* = 0.04; stimulant use disorder: 26.0%, *p* < 0.001; and alcohol use disorder: 4.3%, *p* = 0.003, respectively). Notably, approximately one third of pregnancies with a documented overdose were among women who reported experiencing IPV (34.1%), homelessness or housing instability (35.6%), or incarceration (33.3%) during pregnancy; these proportions were significantly higher than those observed among pregnancies without a documented overdose (IPV: 11.9%, *p* < 0.001; homelessness or housing instability: 19.6%, *p* = 0.008; and incarceration: 16.4%, *p* = 0.002, respectively).

**TABLE 1:** Characteristics of women receiving medication for opioid use disorder (MOUD) by overdose experience during pregnancy—MATernaL and Infant clinical NetworK (MAT‐LINK), 2014–2021.

	Overdose	No overdose	
	*N* (%)	*N* (%)	*p* value
Total^a^	46 (1.2)	3636 (98.8)	
Number of overdoses per pregnancy: Range (median)	1–4 (1)		
Age during pregnancy			0.47
<25 years	8 (17.4)	629 (17.3)	
25–29 years	21 (45.7)	1298 (35.7)	
30–34 years	10 (21.7)	1131 (31.1)	
≥35 years	7 (15.2)	578 (15.9)	
Race and ethnicity			**0.01**
Hispanic/Latina	–	1081 (30.2)	
Non‐Hispanic Black or African American	–	144 (4.0)	
Non‐Hispanic White	35 (77.8)	2223 (62.2)	
Non‐Hispanic Other Race^b^	–	128 (3.6)	
Urban–rural classification during pregnancy			0.37
Urban	40 (88.9)	3290 (91.4)	
Rural	5 (11.1)	308 (8.6)	
Insurance status at delivery			N/A
Public	42 (91.3)	3136 (87.0)	
Private	–	393 (10.9)	
Other (includes no insurance)	–	74 (2.1)	
Number of MOUD treatment encounters during pregnancy			0.06
One encounter only	–	610 (16.8)	
Two or more encounters	43 (93.5)	3026 (83.2)	
Other substance use disorders during pregnancy^c^			
Alcohol use disorder	6 (13.3)	148 (4.3)	**0.003**
Tobacco/nicotine use disorder	35 (77.8)	2168 (62.5)	**0.04**
Cannabis use disorder	11 (24.4)	530 (15.3)	0.09
Stimulant use disorder	24 (53.3)	901 (26.0)	**<0.001**
Psychosocial factors^c^			
Intimate partner violence during pregnancy	15 (34.1)	385 (11.9)	**<0.001**
Homelessness or housing instability during pregnancy	16 (35.6)	642 (19.6)	**0.008**
Incarceration during pregnancy	15 (33.3)	537 (16.4)	**0.002**

*Note*: Cell sizes less than 5 have been suppressed, as indicated by the “–” symbol.

Bold typeface indicates statistical significance at *p* < 0.05.

^a^ Some characteristics do not add up to the column totals due to missingness. There were 61 (1.7%) observations missing data on race and ethnicity, 39 (1.1%) observations missing data on urban–rural classification during pregnancy, 33 (0.9%) observations missing data on insurance status at delivery, 169 (4.6%) observations missing data on alcohol use disorder, 170 (4.6%) observations missing data on tobacco/nicotine use disorder, 170 (4.6%) observations missing data on cannabis use disorder, 169 (4.6%) observations missing data on stimulant use disorder, 391 (10.6%) observations missing data on intimate partner violence during pregnancy, 369 (10.0%) observations missing data on homelessness during pregnancy, and 367 (10.0%) observations missing data on incarceration during pregnancy.

^b^ Includes women who identified as American Indian or Alaska Native, Asian, Native Hawaiian or other Pacific Islander, or multiracial.

^c^ Not mutually exclusive.

Overall, 93.5% of pregnancies with a documented overdose and 83.2% of pregnancies without a documented overdose were among women who had at least two encounters with the healthcare system in which MOUD was prescribed or administered.

**FIGURE 3:**
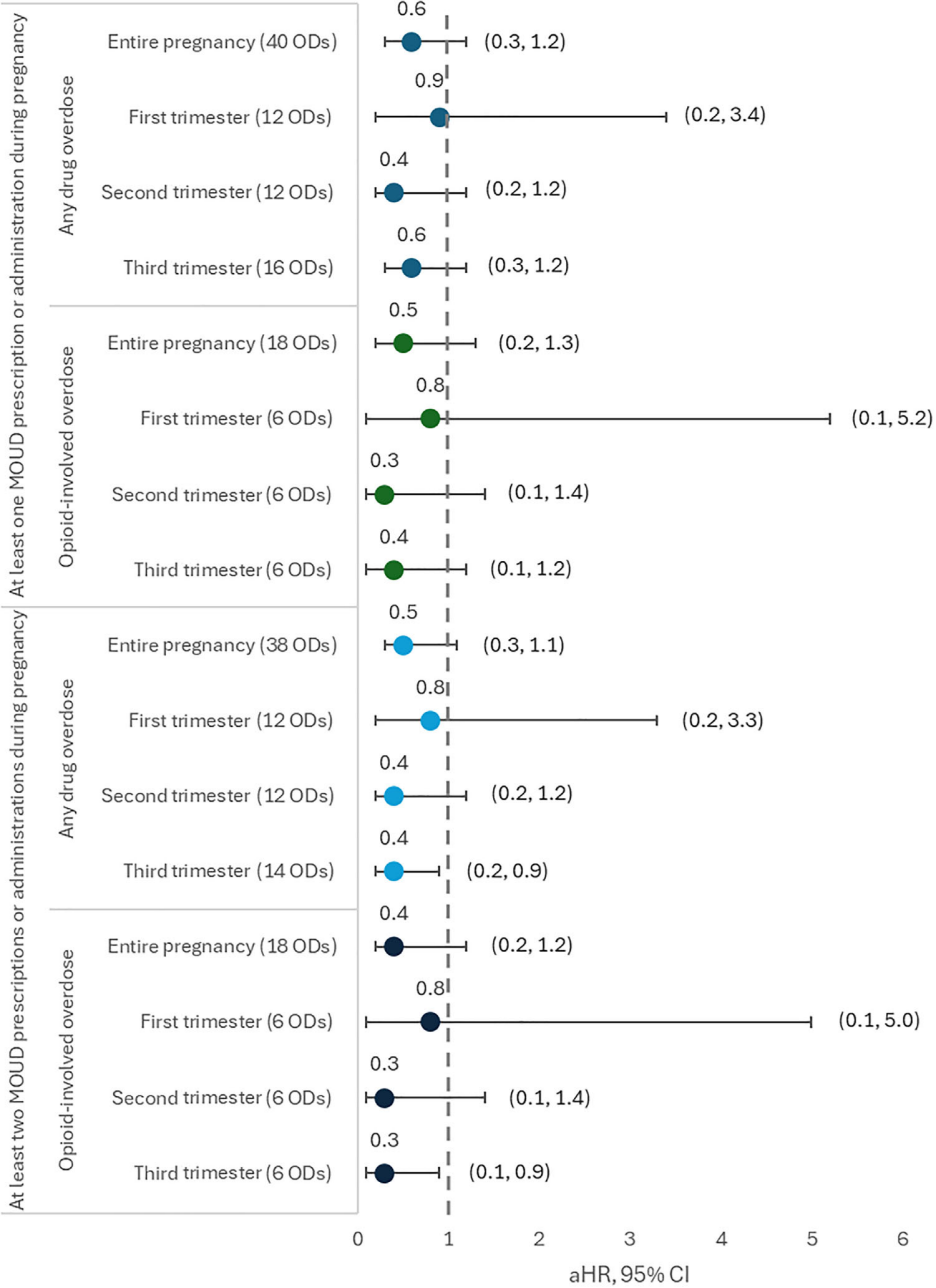
Adjusted hazard ratios (aHR)^*^ and 95% confidence intervals for time to overdose during pregnancy among women prescribed buprenorphine (with or without naloxone) compared with methadone—MATernaL and Infant clinical NetworK (MAT‐LINK), 2014–2021. MOUD, medication for opioid use disorder; ODs, overdose events. ^*^All models were adjusted for the following covariates: maternal age, race and ethnicity, clinical site, homelessness or housing instability, and incarceration during pregnancy.

Among pregnancies with at least one healthcare encounter in which MOUD was prescribed or administered, after controlling for age, race and ethnicity, clinical site, homelessness or housing instability, and incarceration, no differences were observed in overdose across the entire pregnancy among women prescribed buprenorphine compared to methadone (any drug overdose: aHR, 0.6; 95% CI, 0.3–1.2; opioid‐involved overdose: aHR, 0.5; 95% CI, 0.2–1.3; Figure 3). Similarly, among pregnancies with at least two healthcare encounters in which MOUD was prescribed or administered, no differences were observed between buprenorphine and methadone (any drug overdose: aHR, 0.5; 95% CI, 0.6, 0.3–1.1; opioid‐involved overdose: aHR, 0.4; 95% CI, 0.2–1.2) across the entire pregnancy. In the third trimester, among pregnancies with at least two healthcare encounters in which MOUD was prescribed or administered, women receiving buprenorphine were less likely than those receiving methadone to have any overdose (aHR, 0.4; 95% CI, 0.2–0.9) or an opioid‐involved overdose (aHR, 0.3; 95% CI, 0.1–0.9). In all other trimester‐specific analyses, no differences in overdose were observed (Figure 3).

## 4 DISCUSSION

Both buprenorphine and methadone are effective in managing OUD and reducing the risk of experiencing an overdose in the general US population [23]. This analysis found that over the course of pregnancy, there were no differences in nonfatal overdose among women who received buprenorphine versus methadone. In trimester‐specific models, in the third trimester, women with at least two MOUD prescriptions or administrations during pregnancy were less likely to experience an overdose if they received buprenorphine as compared with methadone.

The proportion of pregnancies with an overdose in this analysis (1.2%) is consistent with the literature (2.3%) [9]. In line with previous studies [3, 24, 25], the majority of reported overdoses involved opioids; however, it is worth noting that almost 40% of pregnancies with an overdose did not have reported opioid involvement in the overdose, reflecting a high degree of polysubstance use in this cohort of pregnancies affected by OUD. While 41.3% of pregnancies with an overdose had an overdose involving heroin, only 17.4% reported fentanyl involvement, despite fentanyl being the dominant opioid driving overdose deaths currently [1]. It is likely that fentanyl‐involved overdoses were underreported in this cohort due to the shift in fentanyl dominance in the illicit opioid supply and increased awareness of fentanyl use that occurred later in the study period, as well as the lack of clinical testing for fentanyl earlier in the study period [26, 27, 28].

Significant differences were observed in the characteristics of pregnancies with an overdose compared to those without a reported overdose, particularly with regard to experiencing IPV, homelessness or housing instability, and incarceration, which is consistent with the general US population [29, 30]. These complications during pregnancy might contribute to increased mental health strain, substance use, and delays or interruptions in accessing prenatal care or MOUD [31, 32, 33, 34, 35, 36, 37, 38], which can all predispose women to overdose [7, 9, 23]. Evidence‐based strategies for engagement in addiction care in the perinatal period, such as integrated and collaborative care models, peer support services, patient navigation, telehealth, wrap‐around care services, and incorporating additional support such as doulas, can address the unique barriers these patients experience and enhance continuity of care [39, 40, 41, 42]. Additionally, strategies such as strengthening economic supports and preventing substance use initiation can reduce the likelihood of overdose and experiencing adverse outcomes such as IPV [43, 44].

The findings from this analysis might be affected by important differences in how buprenorphine and methadone are accessed: buprenorphine is prescribed by a clinician and medication is dispensed using outpatient pharmacies, while methadone is administered daily via federally certified opioid treatment programs (OTPs) or in some cases offered as take‐home doses at the program's discretion. As pregnancy progresses, women might find it more difficult to access OTPs, increasing their risk for substance use and overdose. Stigma from methadone and buprenorphine providers has also been reported by women with OUD during pregnancy, and this might create additional barriers for women accessing methadone via daily visits with an OTP as the pregnancy progresses [45]. There are also fundamental differences in the medications themselves and as such there might be differences in the patient populations receiving buprenorphine versus methadone. For instance, patients with fentanyl dependence might be more likely to overdose given the higher potency of fentanyl compared with heroin and other opioids [46]. These patients might also be more likely to be initiated on methadone rather than buprenorphine due to a lengthier abstinence time required before starting on buprenorphine as well as reports of buprenorphine‐precipitated withdrawal from fentanyl [47, 48, 49]. Clinicians might also start patients on buprenorphine, then switch to methadone if the patient is unable to complete induction or maintain adherence [4]. According to a previous study of MOUD patterns in the MAT‐LINK cohort, 42.0% of pregnancies in the MAT‐LINK MOUD cohort were prescribed methadone, 42.5% buprenorphine monoproduct, and 28.2% buprenorphine–naloxone; however, more pregnancies with methadone were among women with public insurance coverage (90.5%) or living in urban areas (87.3%) compared with buprenorphine monoproduct (77.6% and 79.1%, respectively) or buprenorphine–naloxone (81.7% or 78.4%, respectively), reflecting some of the underlying differences in the patient population receiving methadone versus buprenorphine [16]. Additionally, the authors noted that the median gestational age at MOUD initiation in the MAT‐LINK cohort was 20 weeks, while the median gestational age at prenatal care initiation was 15 weeks, providing additional context for the third‐trimester findings in this analysis [16]. In sum, we hypothesize that women receiving methadone in the third trimester in this cohort might have experienced additional challenges in achieving MOUD stability. This might have increased the likelihood of overdose among women receiving methadone as compared with buprenorphine in the third trimester. It is also possible that unmeasured factors influenced the third‐trimester findings, as potentially confounding factors such as years of opioid dependence might influence the type of MOUD pregnant women are offered [50]. Such factors, which were unable to be included in the model, might also explain why all effect estimates from this analysis were protective, though most were not significant.

Regardless, it is critical that there were no differences between buprenorphine and methadone in overdose over the entire pregnancy, as this affords patients and clinicians the flexibility to choose the medication that works best for the individual patient. During MOUD induction and as pregnancy progresses, it is important to continue monitoring the needs of patients, and it might be reasonable to consider switching patients to a different MOUD if they are having difficulty with MOUD adherence, continued opioid cravings, or achieving abstinence [4]. Additionally, metabolic changes during pregnancy might require MOUD dose changes (e.g., dose increases or dosing more than one time per day) to effectively manage opioid cravings, especially during the third trimester [4].

Clinicians providing care to women with OUD during pregnancy can intervene in overdose prevention not only by ensuring continued access to and monitoring of MOUD, but also via overdose education and naloxone access, supplemental support services, and assistance with care navigation [39, 42]. Additionally, facilitated access to community‐based programs such as syringe services programs that provide overdose education and naloxone distribution are key strategies to reduce overdose morbidity and mortality [24, 40, 51].

This analysis adds to a growing body of literature examining the impacts of MOUD during pregnancy. Women with OUD during pregnancy might differ in their ability to access certain MOUD regimens based on several factors, including the accessibility of OTPs that administer or distribute methadone [52] and pharmacies with buprenorphine in stock [53], health insurance coverage [54], living in urban versus rural areas [54], incarceration [55, 56], and experiencing homelessness [57]. Future studies can examine how these differences impact the timing of MOUD receipt, continuity of MOUD, changes in MOUD dosing, and overdose during pregnancy. Additionally, given that MOUD discontinuation and risk of fatal overdose are high during the postpartum period [6, 58], studies examining the impact of MOUD on postpartum overdose timing and frequency are of value.

A strength of this analysis is the robust and longitudinal nature of the data, which provides unique insight into the variability of MOUD and overdose during pregnancy. Another strength is the ability to examine exposures and outcomes by trimester as well as throughout pregnancy. However, this analysis is subject to several limitations. First, overdose events might be underreported since data were collected from an EHR. This is a comprehensive approach that includes gathering data from procedure and diagnosis codes as well as clinical notes and has greater accuracy in identifying overdoses when compared to ICD‐9/10‐CM codes alone [17]. It is therefore less likely that pregnancies in this analysis were falsely identified as having experienced an overdose; however, it is probable that some overdose events that occurred outside of healthcare system intervention were missed [59], particularly if overdose reversal occurred in a community setting or through an emergency medical service response and follow‐up care at a health system was not sought. Results are also reflective only of women with OUD during pregnancy who were receiving MOUD and are limited to encounters that occurred at six clinical sites, which might have biased results away from overdoses that did not involve opioids and might not reflect overdose patterns throughout the United States. As discussed earlier, there are differences in how methadone and buprenorphine are accessed and therefore in the populations that receive them. Additionally, MOUD as documented in the EHR might not correspond to prescription dispensing or actual use by the patient as prescribed. In this analysis, buprenorphine monoproduct and buprenorphine–naloxone were combined to maintain a robust exposure group, potentially diluting the relationship of each buprenorphine product individually on overdose. It should also be noted that access to buprenorphine may have changed over time during the study period, especially following expanded prescribing flexibilities for buprenorphine and methadone during the COVID‐19 pandemic [60] and the release of the 2020 national practice guideline for OUD treatment from the American Society of Addiction Medicine, which included a consensus by the guideline committee that buprenorphine–naloxone is safe and effective for use during pregnancy [61]. However, these changes occurred during the final 2 years of the cohort and might not have greatly influenced the overall analysis results. In addition, all reported overdoses in this analysis were nonfatal; fatal overdoses that occurred during pregnancy might not have been captured in MAT‐LINK since the inclusion criteria was limited to women with a known pregnancy outcome. These factors taken together limit the generalizability of the overdose rate and modeling results.

## 5 CONCLUSION

MOUD during pregnancy is a critical component of care management to reduce morbidity and mortality associated with OUD. In this longitudinal analysis, no differences in overdose during pregnancy were observed between buprenorphine and methadone; in an analysis of the third trimester only and among pregnancies with at least two MOUD prescriptions or administrations, buprenorphine was associated with a reduced likelihood of overdose compared with methadone. It is important that women with OUD during pregnancy are initiated on MOUD as early as possible and that continuous monitoring occurs throughout pregnancy, paying special attention to disruptions and changes during the third trimester that might increase risk for overdose. Ensuring that women with OUD have access to clinical‐ and community‐based overdose education and naloxone throughout pregnancy and into the postpartum period can help reduce the number of overdoses observed in this population.

## AUTHOR CONTRIBUTIONS

**Amy Board**: Conceptualization; methodology; formal analysis; writing—original draft. **Alana M. Vivolo‐Kantor**: Conceptualization; methodology; writing—review & editing. **Shin Y. Kim**: Conceptualization; methodology; writing—review & editing. **Shawn A. Thomas**: Validation; writing—review & editing. **Emmy L. Tran**: Conceptualization; methodology; writing—review & editing. **Mishka Terplan**: Conceptualization; writing—review & editing. **Pilar M. Sanjuan**: Data Curation; project administration; writing—review & editing. **Marcela C. Smid**: Data Curation; project administration; writing—review & editing. **Tanner Wright**: Data Curation; project administration; writing—review & editing. **Neil S. Seligman**: Data Curation; project administration; writing—review & editing. **Patrick D. Schneider**: Data Curation; project administration; writing—review & editing. **Autumn Davidson**: Data Curation; project administration; writing—review & editing. **Michelle L. Henninger**: Data Curation; project administration; writing—review & editing. **Elisha M. Wachman**: Data Curation; project administration; writing—review & editing. **Kara M. Rood**: Data Curation; project administration; writing—review & editing. **Luigi Garcia Saavedra**: Data Curation; project administration; writing—review & editing. **Judette Louis**: Data Curation; project administration; writing—review & editing. **Diane Morse**: Data Curation; project administration; writing—review & editing. **Julie Shakib**: Data Curation; project administration; writing—review & editing. **Kelley A. Saia**: Data Curation; project administration; writing—review & editing. **Kathryn Miele**: Conceptualization; methodology; writing—review & editing; supervision.

## CONFLICT OF INTEREST STATEMENT

Amy Board, Alana M. Vivolo‐Kantor, Shin Y. Kim, Emmy L. Tran, Shawn A. Thomas, Mishka Terplan, Patrick D. Schneider, Autumn Davidson, Michelle L. Henninger, Elisha M. Wachman, Kara M. Rood, Luigi Garcia Saavedra, Judette Louis, Diane Morse, Julie Shakib, and Kelley A. Saia declare no conflicts of interest. Unrelated to this work, Marcela C. Smid's institution receives research funds supporting her time from Gilead Inc and Koko Medical and previously received funds from Organon Inc. Tanner Wright reports funding from Duke University for a National Institutes of Health–funded project on Eating, Sleeping, Consoling for Neonatal Opioid Withdrawal Syndrome (ESC‐NOW): A Function‐Based Assessment and Management Approach; the National Institute on Drug Abuse for CADENCE (Continuous and Data‐driven Care); the American Academy of Pediatrics for work as a subject matter expert on maternal–infant health and opioid use; and the National Advanced Practice and Low Risk Neonatal Nurses Conferences for presentations on neonatal opioid withdrawal syndrome. Pilar M. Sanjuan reports funding as a principal investigator from the National Institute on Alcohol Abuse and Alcoholism for Project REACT (neural underpinnings of emotional regulation and drinking to cope); the National Institute on Drug Abuse for Project Vida (prenatal opioid use disorder and posttraumatic stress disorder); and the National Institute of Mental Health for Project Raptor (attention and post‐traumatic stress disorder) and as a coinvestigator from the National Institute on Alcohol Abuse and Alcoholism on Project AsCENd (neuroimaging of child attention development); the National Institute on Drug Abuse Helping to End Addiction Long‐Term Initiative for Integrative Management of Chronic Pain and OUD for Whole Recovery (pain and prenatal opioid use) and for the HEALthy Brain and Child Development study; and relationships with New Mexico Postpartum Support International as a board member, the New Mexico Perinatal Collaborative as a board member, and Drexel University as a paid consultant. Neil S. Seligman reports funding from PreTel Inc. and Mae Stone Goode for work not related to this report; Natera for consulting fees; UpToDate for writing; and various legal reviews. Kathryn Miele reports travel arrangements and conference registration when presenting at meetings with the American College of Obstetricians and Gynecologists, Association of Women's Health, Obstetric, National Association of Nurse Practitioners in Women's Health, and Neonatal Nurse, Infectious Diseases Society for Obstetrics and Gynecology, and Society for Maternal‐Fetal Medicine.

## Supporting information



Supporting Information

## Data Availability

A limited MAT‐LINK dataset is available through the National Center for Health Statistics' Research Data Center (https://www.cdc.gov/rdc/other‐restricted‐data/mat‐link.html). The available data focus on pregnant woman, infant, and child health outcomes associated with medication for opioid use disorder during pregnancy.
