# Supplementary material for: Examining buprenorphine compared to methadone for likelihood of overdose during pregnancy, 2014–2021
Source: Pregnancy (Hoboken). 2026 Apr 7;2(3):e70276. doi: 10.1002/pmf2.70276 (PMC13344214; doi:10.1002/pmf2.70276)
Supplement: Supplementary file 1 [file PMF2-2-e70276-s001.docx]

**SUPPLEMENTAL DIGITAL CONTENT**

Supplemental Table 1: International Statistical Classification of Diseases and Related Health Problems, 9^th^ and 10^th^ Revision, Clinical Modification (ICD-9/10-CM) Codes Used to Determine Diagnosis of Opioid Use Disorder During Pregnancy in the MATernaL and Infant clinical NetworK (MAT-LINK)

| **Code and category** | **Description** |
| --- | --- |
| **ICD-10-CM** | |
| F11 | Opioid-related disorders |
| F11.1X* | Opioid abuse |
| F11.2X* | Opioid dependence |
| F11.9X* | Opioid use, unspecified |
| F11.10 | Opioid abuse, uncomplicated |
| F11.11 | Opioid abuse, in remission (only for women receiving MOUD during pregnancy) |
| F11.12X* | Opioid abuse with intoxication |
| F11.13 | Opioid abuse with withdrawal |
| F11.14 | Opioid abuse with opioid-induced mood disorder |
| F11.15X* | Opioid abuse with opioid-induced psychotic disorder |
| F11.18X* | Opioid abuse with other opioid-induced disorder |
| F11.19 | Opioid abuse with unspecified opioid-induced disorder |
| F11.21 | Opioid dependence, in remission (only used for women receiving MOUD during pregnancy) |
| F11.22X* | Opioid dependence with intoxication |
| F11.23 | Opioid dependence with withdrawal |
| F11.24 | Opioid dependence with opioid-induced mood disorder |
| F11.25X* | Opioid dependence with opioid-induced psychotic disorder |
| F11.28X* | Opioid dependence with other opioid-induced disorder |
| F11.29 | Opioid dependence with unspecified opioid-induced disorder |
| F11.92X* | Opioid use, unspecified with intoxication |
| F11.93 | Opioid use, unspecified with withdrawal |
| F11.94 | Opioid use, unspecified with opioid-induced mood disorder |
| F11.95X* | Opioid use, unspecified with opioid-induced psychotic disorder |
| F11.98X* | Opioid use, unspecified with other specified opioid-induced disorder |
| F11.99 | Opioid use, unspecified with unspecified opioid-induced disorder |
| O99.320 | Drug use complicating pregnancy, unspecified trimester |
| O99.321 | Drug use complicating pregnancy, first trimester |
| O99.322 | Drug use complicating pregnancy, second trimester |
| O99.323 | Drug use complicating pregnancy, third trimester |
| O99.324 | Drug use complicating childbirth |
| O99.325 | Drug use complicating the puerperium |
| T40.0X* | Poisoning by, adverse effect of, and underdosing of opium |
| T40.1X* | Poisoning by and adverse effect of heroin |
| T40.2X* | Poisoning by, adverse effect of, and underdosing of other opioids |
| T40.3X* | Poisoning by, adverse effect of, and underdosing of methadone |
| T40.4XX* | Poisoning by, adverse effect of, and underdosing of other synthetic narcotics |
| T40.6XX* | Poisoning by, adverse effect of, and underdosing of other and unspecified narcotics |
| **ICD-9-CM** | |
| 648.30 | Drug dependence of mother, unspecified as to episode of care or not applicable |
| 304.00 | Opioid-type dependence, unspecified |
| 304.01 | Opioid-type dependence, continuous |
| 304.02 | Opioid-type dependence, episodic |
| 304.03 | Opioid-type dependence, in remission (only for women receiving MOUD during pregnancy) |
| 304.70 | Combinations of opioid-type drug with any other drug dependence, unspecified |
| 304.71 | Combinations of opioid-type drug with any other drug dependence, continuous |
| 304.72 | Combinations of opioid-type drug with any other drug dependence, episodic |
| 304.73 | Combinations of opioid-type drug with any other drug dependence, in remission (only for women receiving MOUD during pregnancy) |
| 305.50 | Opioid abuse, unspecified |
| 305.51 | Opioid abuse, continuous |
| 305.52 | Opioid abuse, episodic |
| 305.53 | Opioid abuse, in remission (only for women receiving MOUD during pregnancy) |
| 965.0X* | Poisoning by analgesics, antipyretics, and antirheumatics |
| E850.0 | Accidental poisoning by heroin |
| E850.1 | Accidental poisoning by methadone |
| E850.2 | Accidental poisoning by other opiates and related narcotics |
| E935.0 | Heroin causing adverse effects in therapeutic use |
| E935.1 | Methadone causing adverse effects in therapeutic use |
| E935.2 | Other opiates and related narcotics causing adverse effects in therapeutic use |

Abbreviation: MOUD = medication for opioid use disorder.
* X is a placeholder character to indicate inclusion of all valid subcategories and codes.

Supplemental Figure 1: Flow Diagram of Pregnancies Included in the Analysis

Pregnancies without MOUD:

1,617

Pregnancies missing MOUD trimester data: 11

Pregnancies excluded due to missingness:

Missing or incorrect date of delivery or gestational age: 19

Missing date of overdose or overdose occurred prior to the start of pregnancy: 15

Dropped records from a site with greater than 60% missingness for key analytic variables: 196

Pregnancies included in the analysis: 3,682

Pregnancies with at least one EHR-documented MOUD: 3,912

Abbreviations: EHR = electronic health record, MOUD = medication for opioid use disorder

All MAT-LINK pregnancies:

5,540

Supplemental Table 2: List of Opioid and Non-Opioid Substances Involved in an Overdose During Pregnancy Documented in the MATernaL and Infant clinical NetworK (MAT-LINK)

| **Opioids** | **Non-Opioid Substances** |
| --- | --- |
| Alfentanil | Alcohol |
| Alphaprodine | Barbiturates |
| Buprenorphine | Cocaine |
| Butorphanol | Gabapentinoids |
| Codeine | Hallucinogens |
| Dezocine | Inhalants |
| Dihydrocodeine | Kratom |
| Fentanyl | Loperamide |
| Heroin | Marijuana |
| Hydrocodone | Methamphetamine |
| Hydromorphone | Prescription stimulants |
| Levorphanol |  |
| Meperidine |  |
| Methadone |  |
| Morphine |  |
| Nalbuphine |  |
| Opium |  |
| Oxycodone |  |
| Oxymorphone |  |
| Pentazocine |  |
| Remifentanil |  |
| Sufentanil |  |
| Tapentadol |  |
| Tramadol |  |

Note: Free text fields were used to capture any other substances not listed above.
